# A Study on the Thermomechanical Reliability Risks of Through-Silicon-Vias in Sensor Applications

**DOI:** 10.3390/s17020322

**Published:** 2017-02-09

**Authors:** Shuai Shao, Dapeng Liu, Yuling Niu, Kathy O’Donnell, Dipak Sengupta, Seungbae Park

**Affiliations:** 1 Department of Mechanical Engineering, State University of New York at Binghamton, P.O. Box 6000, Binghamton, NY 13902, USA; yniu1@binghamton.edu (Y.N.); sbpark@binghamton.edu (S.P.); 2 Analog Devices, Inc., Wilmington, MA 01887, USA; kathy.odonnell@analog.com (K.O.); dipak.sengupta@analog.com (D.S.)

**Keywords:** MEMS packaging, optical sensor, TSV, reliability, finite element analysis (FEA), fracture mechanics

## Abstract

Reliability risks for two different types of through-silicon-vias (TSVs) are discussed in this paper. The first is a partially-filled copper TSV, if which the copper layer covers the side walls and bottom. A polymer is used to fill the rest of the cavity. Stresses in risk sites are studied and ranked for this TSV structure by FEA modeling. Parametric studies for material properties (modulus and thermal expansion) of TSV polymer are performed. The second type is a high aspect ratio TSV filled by polycrystalline silicon (poly Si). Potential risks of the voids in the poly Si due to filling defects are studied. Fracture mechanics methods are utilized to evaluate the risk for two different assembly conditions: package assembled to printed circuit board (PCB) and package assembled to flexible substrate. The effect of board/substrate/die thickness and the size and location of the void are discussed.

## 1. Introduction

In recent years, TSV technology has reshaped the packaging of electronic devices. By providing a vertical electrical connection passing completely through the silicon die, this technology can help to reduce the length of the connection paths and the size of electronic packages. As the industry demand for performance and miniaturization has increased, three-dimensional (3-D) integration using TSV has drawn attention from industry and academia. The technical community has witnessed a large amount of research and development work in every related area: via etching [1,2] and filling, wafer bonding [3], interconnection [4], thermal management [5], and thermomechanical reliability [6], etc.

However, many technical challenges remain for 3-D packaging. One major challenge for 3-D integrated circuits (IC) is thermal management. The high-density circuits in the chip-stack can generate a large amount of heat due to Joule heating that leads to thermo-mechanical stress and atomic migration, both of which may cause reliability issues [7].

A large variety of 3-D integrated products have come to market, including FPGAs, CMOS image sensors, active pixel sensors, MEMS resonators and accelerometers [8]. MEMS is still the one of pioneers of development and application of 3-D/TSV technologies. This is because, compared to the devices with high power density, it is relatively easier to implement TSV-based 3-D packaging technology in the devices that have lower heat-generation such as memories, silicon interposers (“2.5D packaging”), and microelectromechanical system (MEMS) products, etc. In addition, MEMS devices usually do not have a large number of I/Os and typically have only two or three dies if there is only one sensor in the package. There are many types of MEMS devices and optical sensors that have benefitted from TSV technology in reducing package size, simplifying fabrication process, and saving cost. For example, Zoschke et al. used a silicon interposer with Cu-filled TSVs as the substrate of a MEMS resonator in vacuum packaging in a wafer level process [9]. Ramm et al. developed a TSV 3-D integrated tire pressure monitoring system [10]. The pressure sensor die and the transceiver die are embedded with metal TSVs to match routing and interconnection layer [10]. MEMS resonator [11] and radio frequency (RF) MEMS switches [12,13] were fabricated combining wafer-level package and TSV connection. Griffin et al. developed a thermoelastic ultrasonic actuator using integrated poly-Si TSVs [14]. Those TSVs with SiO_2_ insulation were used for contacting the driving and sensing circuits on the backside of the MEMS wafer [14]. The TSV noise was found low enough to not adversely affect device performance [14]. Inertia sensors were developed using poly-Si TSVs by Hirama, which were fabricated by etching annular trenches in a heavily-doped silicon substrate to surround silicon pillars [15]. This silicon pillar serves as a TSV conductor and the trench serves as an insulator [15]. A hetero-integration method bonding wafers using TSVs to transfer a silicon on insulator (SOI) layer onto a CMOS wafer was developed [16]. TSVs can eliminate the wire bonds and enable higher interconnection densities with a smaller form factor. In the field of optical sensor, CMOS image sensor is currently a category with the highest production volume utilizing 3-D/TSV technologies [8].

The reliability of interconnections is crucial to the electronic product. During the fabrication and usage, dies together with TSVs experience temperature changes (thermal loading). Because different materials in the package have different coefficients of thermal expansion (CTE), stress can develop in the TSVs either due to the local CTE mismatch between a silicon die and via-filling materials, or due to the global deformation of the package, which is also a result of CTE mismatch.

Many different types of TSVs are being adopted for various applications, and the reliability challenges can be different from case to case. Fully-filled copper TSV has been studied extensively both in experiment and simulation. Suhir studied analytical solutions for stress in TSVs in 3-D packaging [17]. Disc-like vias (height/thickness-to-diameter ratio below 0.25) and rod-like vias (height/thickness-to-diameter ratio above 2.5) were investigated. Protrusion and buckling are the major potential risks for reliability, which is also observed and discussed [18,19]. One way to reduce protrusion risk is using partially-filled copper TSV, which is only studied by limited research. Compared to MEMS sensors, larger and thicker dies are used in optical sensors. Poly-Si TSVs is able to decrease reliability risk to some extent, since there is almost no CTE mismatch. However, potential risk of the voids in the poly Si due to filling defects is a new concern, and there is limited study on it.

In this paper, two types of TSV design are studied via the finite element method: a partially-filled copper TSV and a poly-Si TSV. These two types of TSVs have a similar diameter, although the aspect ratio is different. The influence of the filling polymer material in Cu TSVs on package-level reliability is discussed. Risks of the voids in the poly Si due to filling defects are discussed. The effects of void condition, die thickness, substrate thickness, and substrate material on the poly-Si TSV thermo-mechanical reliability are discussed for sensor application. This paper is composed of 10 chapters. Section 2, Section 3 and Section 4 cover the FEA modeling and discussion on one kind of copper TSVs which is called type A. Section 5, Section 6, Section 7 and Section 8 cover the FEA modeling and discussion on poly-Si TSV which is called type B.

## 2. TSV in MEMS Sensor

Copper TSVs in high-resistance silicon wafers or glass wafers are able to achieve low signal transmission losses. TSVs can be located either in the cap wafer [13] or in the substrate [14]. The MEMS and ASIC die can have side-by-side configuration or stacked configuration if the die size is appropriate. Molding is often used to protect the dies and bonding wires. An alternative approach is to build a cavity package that does not use the mold as the encapsulant but rather uses a lid to provide protection to inside components.

**Figure 1:**
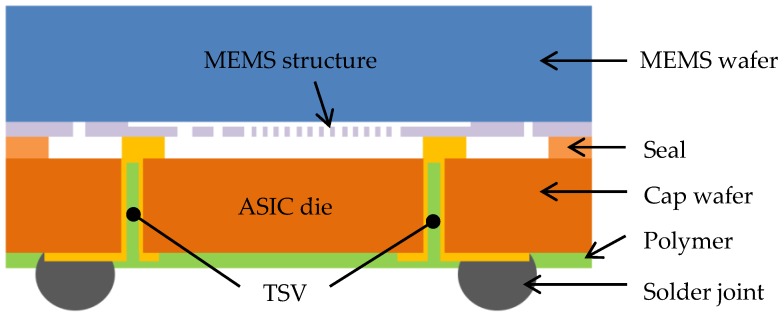
Schematic cross-section image of the MEMS sensor with TSVs through the CMOS IC die.

There are many ways to interconnect dies in an MEMS package if TSV can be fabricated through dies between the MEMS die and ASIC die. Different integration schemes have been discussed in [20]. One of the typical schemes is to flip the MEMS die and create a direct electrical connection to the ASIC using several pairs of nodes that mate with each other. A metal ring is applied on the peripheral regions of the die for hermetic sealing. The TSVs are fabricated through the ASIC die, and the solder bumps are made on the bottom of the ASIC as second-level interconnection. A schematic diagram of this interconnect structure is shown in Figure 1. Cap wafer and MEMS wafer are bonded. TSVs are not necessarily placed to match solder joints. Compared with a conventional packaging scheme, no extra capping is needed for this method, which is a great advantage. In addition, wafer-to-wafer (W2W) bonding becomes possible that can enhance the manufacturing throughput.

**Figure 2:**
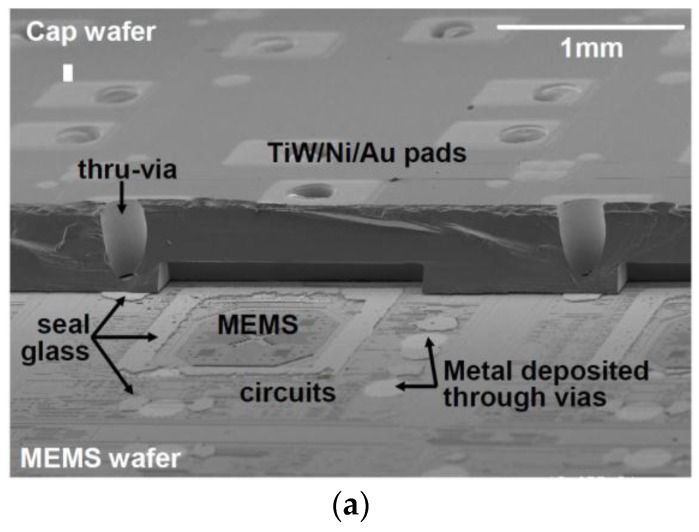

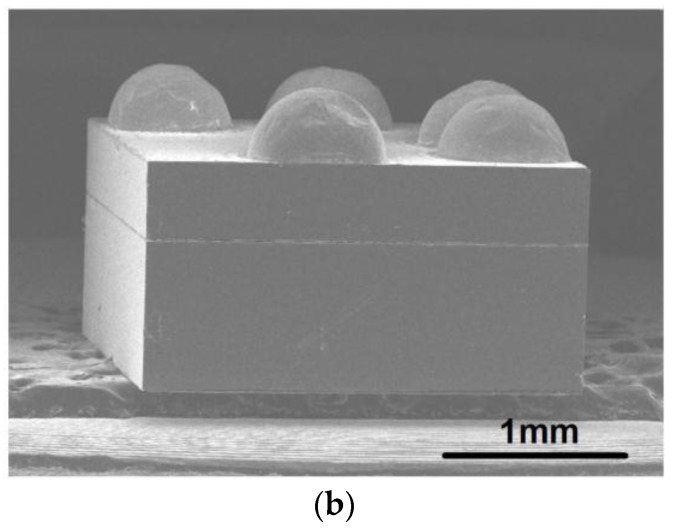
SEM images of MEMS accelerometers with TSVs [21]. (**a**) Bird’s eye view of bonded MEMS wafer and cap wafer with part of cap wafer cleaved away; (**b**) The MEMS accelerometer package after solder-bumping and dicing.

MEMS sensors using TSVs allow significant package size reduction and cost savings. Micromachined MEMS accelerometers were fabricated combining TSV technology and wafer-level-package technology [21]. It has a similar structure as shown in Figure 1, with TSVs through the cap wafer connecting MEMS wafer surface. The TSVs were achieved by two-step deep reactive-ion etching (DRIE) with different masks. Glass frit was used as a bonding and hermetic sealing material. A gap-filling material was used between the bottom of TSVs and the device wafer surface. Figure 2a shows an SEM image after bonding the cap wafer to the MEMS wafer and sputter-deposition of metals. A portion of the cap wafer was intentionally cleaved, in order to show the MEMS structures and circuits, seal glass pattern, and metals deposited TSVs. Each die has five I/Os matching TSVs and metal pads on the cap wafer. Figure 2b shows the accelerometer after solder bumping and dicing. Given the die dimensions of 2.3 mm × 2.3 mm and the thickness of 680 μm, this package size was the same as the die size, much smaller compared to the ceramic package (5 mm × 5 mm × 2 mm) and the plastic package (4 mm × 4 mm × 1.5 mm) [21]. Due to the low density and large diameter of the TSVs, the partially-filling structure is preferred over the fully-filled metal TSV, as they yield lower fabrication cost and lower failure risks [22] which include copper protrusion and buckling due to thermal stress. Therefore our study on Cu TSV followed the integration scheme in Figure 1.

## 3. Finite Element Model for Cu TSV

**Figure 3:**
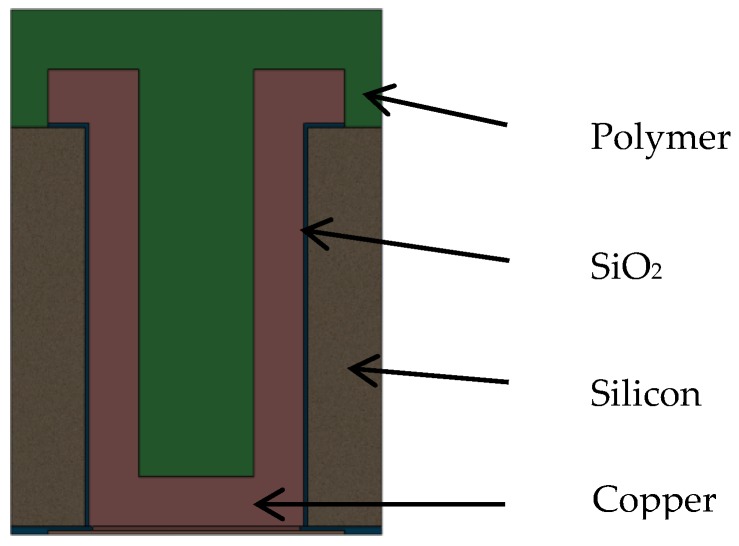
Schematic of TSV structure of Type A.

**Figure 4:**
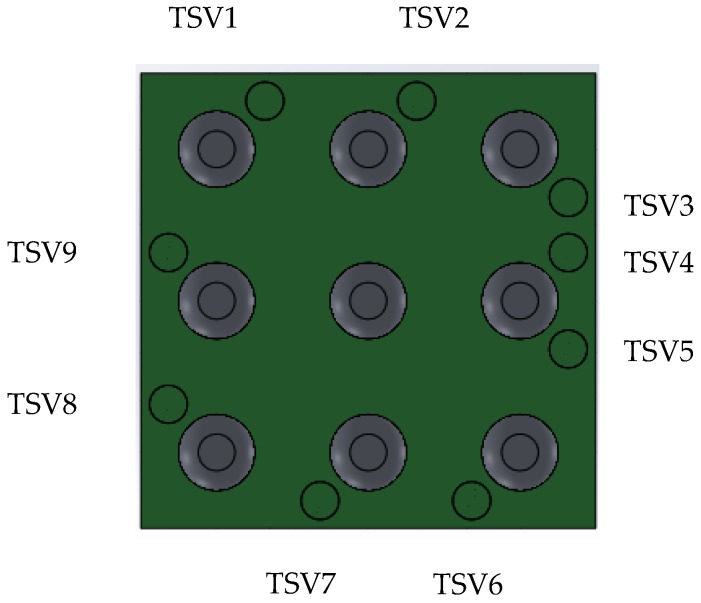
MEMS package with solder joints (view from board side).

A 3-D package-level FEA model, which includes the package with partially-filled copper TSV, solder joints was built. The package in the model was based on Figure 1. Partially-filled TSVs were modeled as shown in Figure 3, called “Type A TSV” in this paper. There are nine TSVs in this package, shown in Figure 4. The form factors of TSVs and packages were from [23,24,25], including package size and thickness, etc. The thickness of the ASIC die was 100 μm. The aspect ratio of the via was 5:3. The TSV copper thickness was referred to the one in [23]. The remaining cavity in the TSV was then filled by polymer, as mentioned previously.

**Figure 5:**
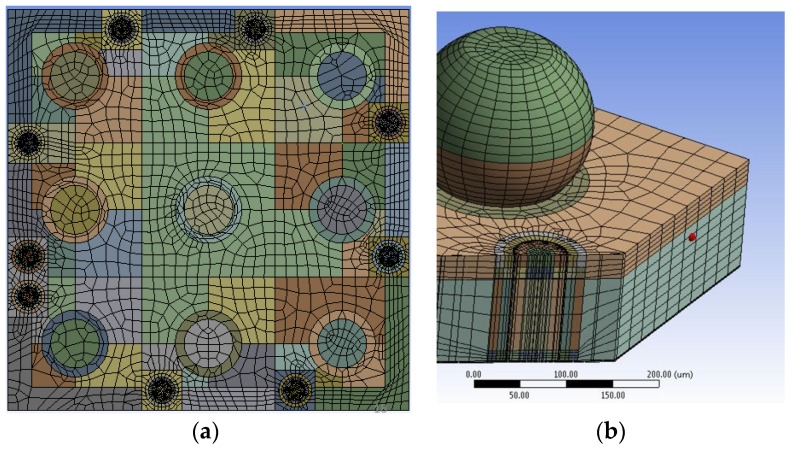
Mesh patterns (**a**) in a top view of MEMS die; (**b**) in a cross-section view of a TSV of Type A (PCB not shown). Various colors indicate different meshing blocks.

In the model, the TSV included an ASIC die, a MEMS die, TSVs, metal sealing rings, solder balls. MEMS structures of accelerometer and gyroscope on the MEMS die surface were neglected in the model. The solder bumps were on the “top” side in Figure 3, and the metal nodes were on the “bottom” side in Figure 3 to make interconnection with the MEMS die. Mesh patterns for package with TSVs of Type A and a solder joint are shown in Figure 5.

Cu fatigue under thermal cycling (between −40 °C and 125 °C) was assumed as the failure mode for this simulation. First by engineering judgment, those corners (in 3-D) are the risky locations. Then ranking the stress level of each possible failure location can help better understanding the thermo-mechanical reliability of this TSV design. This simulation compares stresses in TSV copper at different locations and predict which location is supposed to fail earlier than others. The material properties used in the finite element models are shown in Table 1. 

## 4. Discussion: Reliability Risks for Cu TSV

Reliability is always a major concern for electronic packaging. For the emerging 3-D packaging, the reliability issues of the TSV due to thermal stress are critical. Therefore, identifying the risk sites and studying the influence factors on the risks is necessary in the design stage. Finite element analysis (FEA) has been used for this task. For example, Liu [26] and Lu [27] analyzed the stress and crack driving force with several different via design parameters. However, many different via types have been developed, and their reliability needs to be verified.

**Figure 6:**
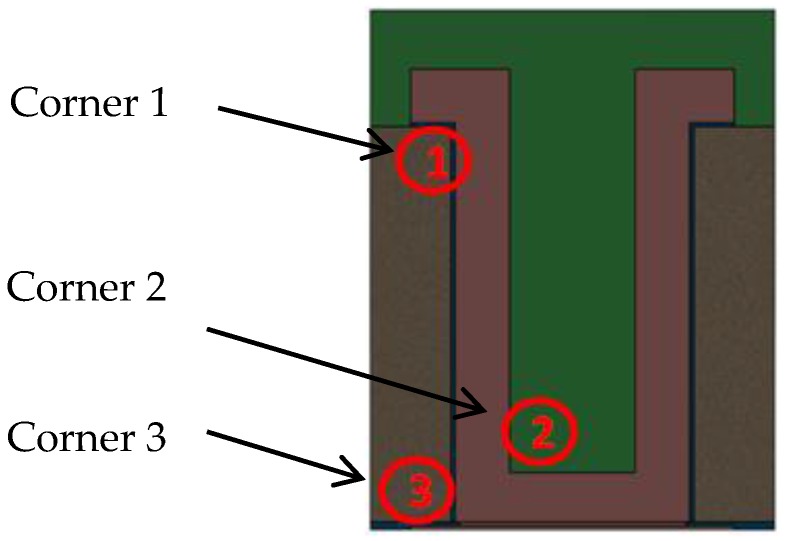
Schematic showing the critical locations of TSV Type A: Corner 1, Corner 2 and Corner 3.

TSV Type A has a half-open structure with a layer of Cu covering the side wall and “bottom” of TSV, and the cavity is filled by polymer. Therefore, the most critical location for the Cu layer will be the corners labeled in Figure 6. The reliability risk can be affected by the material property of the polymer. The height and diameter were kept unchanged in this study.

### Effect of the Polymer Material Properties: CTE and Young’s Modulus

**Figure 7:**
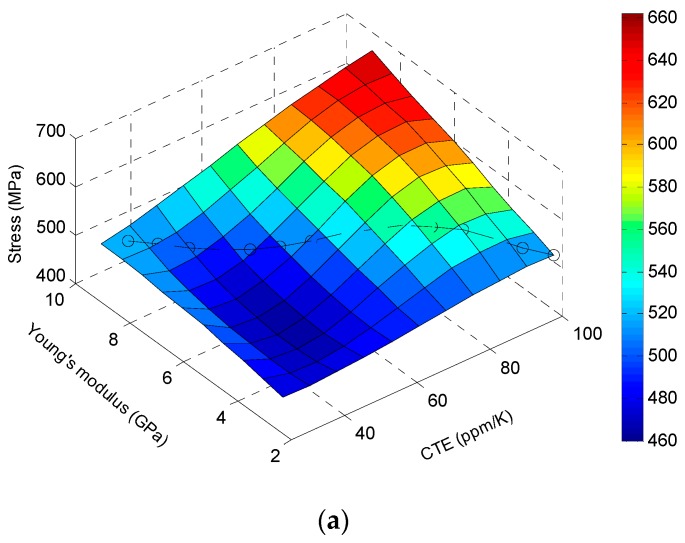

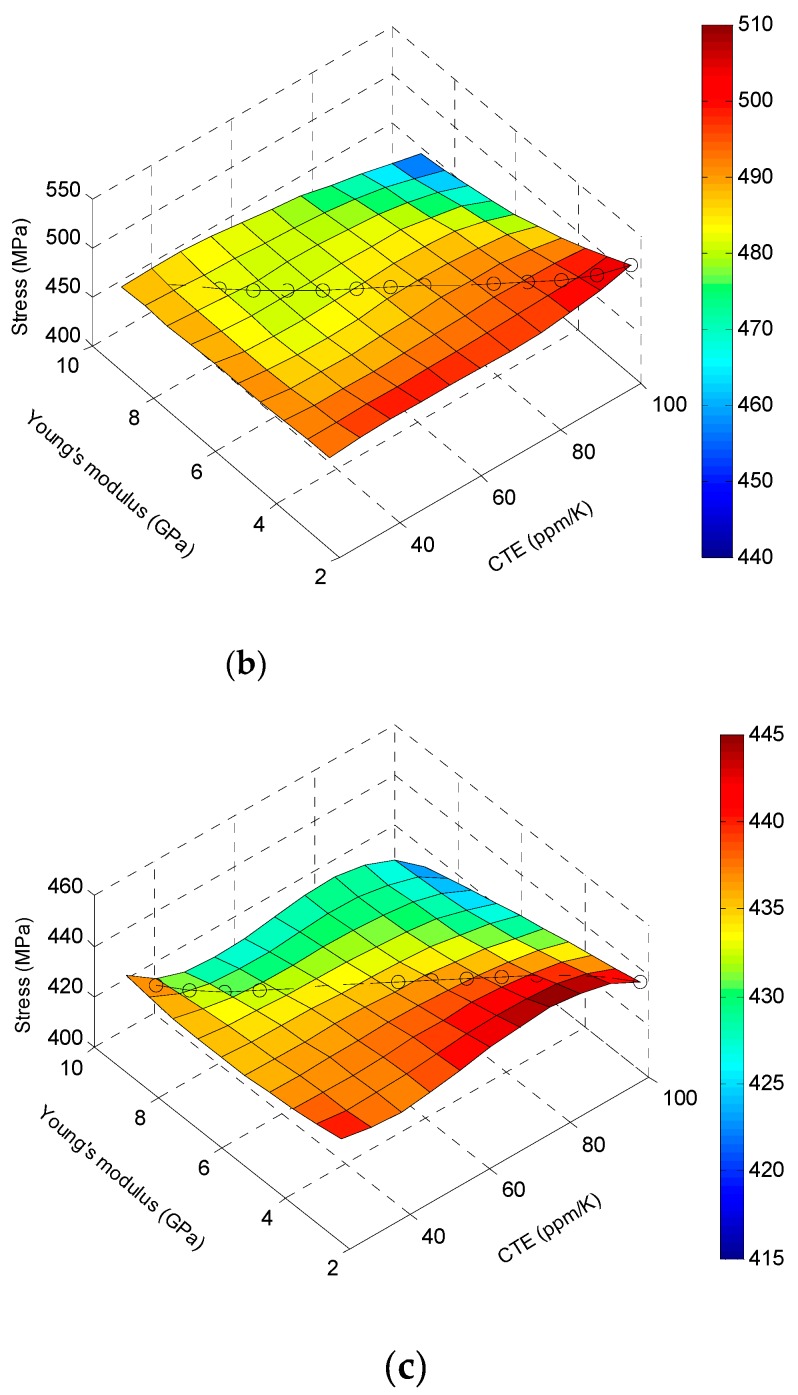
Effect of CTE and Young’s modulus of TSV polymer on TSV stress at three critical locations for Type A. (**a**) Corner #1; (**b**) Corner #2; (**c**) Corner #3.

In order to investigate the effect of polymer material properties on the reliability of the TSV, parametric studies on CTE and Young’s modulus were performed for the TSV model. These two parameters are the key material parameters that affect the thermo-mechanical stresses of the TSV. Equivalent stresses (von Mises stresses) in TSV copper at different corners are obtained in ANSYS Workbench. Figure 7 shows how the TSV stresses vary as CTE and Young’s modulus of the polymer change. Stresses at three corners are plotted separately. CTE range is from 25 ppm/°C to 100 ppm/°C. Young’s modulus range is from 2.3 GPa to 9.2 GPa. Stress data were obtained from ANSYS, and they are plotted after splines interpolation. For Corner #1, it appears that the best combination is low CTE and low modulus so that the TSV stress is low. However, the actual material may not exhibit both advantages at the same time.

**Figure 8:**
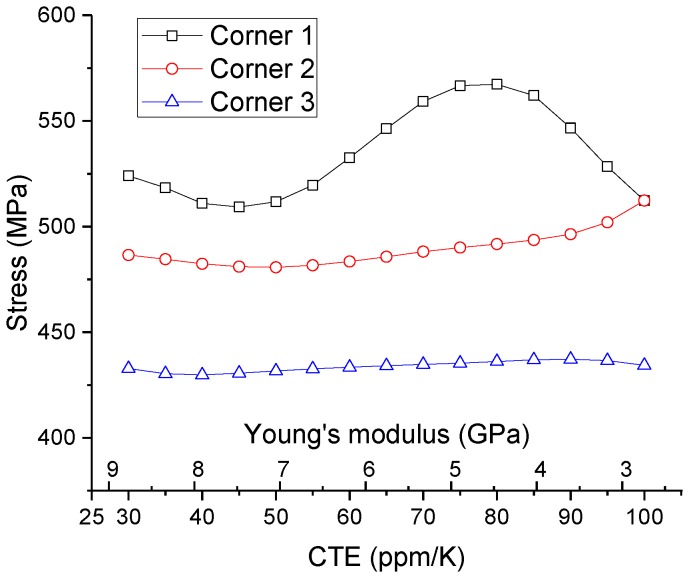
Effect of Young’s modulus on the von Mises stress at the critical locations for TSV Type A.

Practically speaking, a material with low CTE typically has high Young’s modulus (hard) and vice versa. In this sense, in each corner’s plot of Figure 7 one can draw a diagonal line along the surface, which is marked with circles. In fact, it shows how the stresses change correspondingly from one material with CTE of 100 ppm/°C, Young’s modulus of 2.3 GPa to the other material with CTE of 30 ppm/°C, Young’s modulus of 8.7 GPa. These diagonal lines are compared in Figure 8 for those three corners. Figure 8 shows that Corner #1 is the most sensitive to the material change thus has the highest risk out of three corners. Corner #2 and Corner #3 show a slight difference as the polymer material changes. Due to CTE mismatch, the package is supposed to have warpage. Bending stress caused by the warpage has a larger value near the surface of the package than inside the package. One can see that Corner #2 and #3 are inside the package (between MEMS die and ASIC die), while Corner #1 is close to the surface of the package. Material properties (CTE and modulus) of the insulation polymer substantially affect the package warpage. Thus the stress at Corner #1 shows a more evident function of polymer material than stresses at the other two corners.

## 5. Poly-Si TSV for Optical Sensor

Optical sensors are among the earliest products that adopted TSV in volume manufacturing. This interconnection method can eliminate the need for wire bonds and allow higher interconnect density and smaller package size.

The optical sensor we analyzed in this work has a die of 16 mm by 16 mm by 0.5 mm. It has 400 bumps with minimum pitch of 0.5 mm, and 272 TSVs were fabricated to provide interconnection between the optical surface and backside of the die. A redistribution layer is located on the backside.

**Figure 9:**
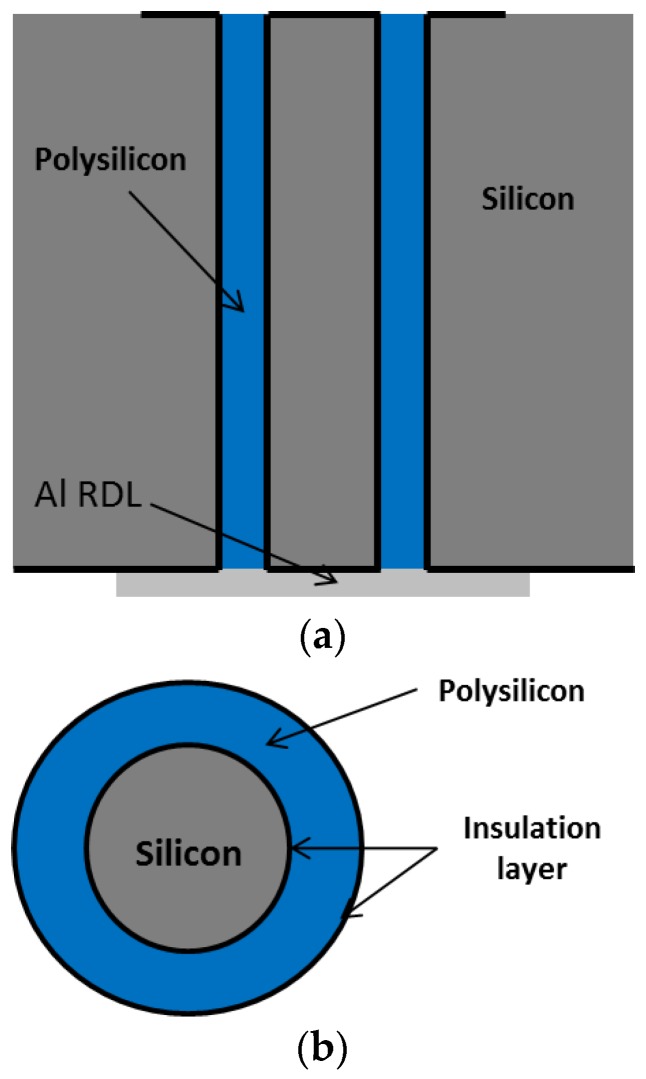
Structure of polycrystalline-silicon-based TSV Type B (**a**) in a cross-section view; (**b**) in a top view.

The structure of the TSV used in this optical sensor is shown in Figure 9 and will be referred to as Type B hereafter. It has 500 μm height. The filling material for this via is polycrystalline silicon that fills an annular gap, and the center of the TSV remains Si. If using the gap distance and the die thickness, the aspect ratio of TSV filling (poly-Si) is 25:1. Material properties of poly-Si in Ref. [28] are used. Thermal silicon oxide was used for isolation. The process for this type of TSV was described in [29,30].

Though the single crystalline silicon and polycrystalline silicon have differences in several material properties, they have very similar CTE, meaning that the thermal stress caused by the CTE mismatch between the Si die and filling material may not be an issue for this via type. There are some stresses due to the filling process [29,30], yet as long as the TSV can be successfully fabricated, we do not think this stress would be the major reliability concern. However, the cross-section image does show that there could be some defects such as seam void in the poly-Si [29,30]. Though a stand-alone die is unlikely to fail even with the seam void in poly-Si, when assembled to PCB, the CTE mismatch between die and PCB may introduce deformation and stress to the die, and stress concentration will happen near the tip of the seam void. Large die thickness potentially leads to higher risk because it may lead to longer void due to the technical difficulty in filling. Solder joint failure may also be a concern for this package and there have been abundant literatures on the quality and reliability of solder joints [31,32,33,34,35]. However, solder joint reliability is not covered here.

## 6. Finite Element Model for Poly-Si TSV in Optical Sensor

**Figure 10:**
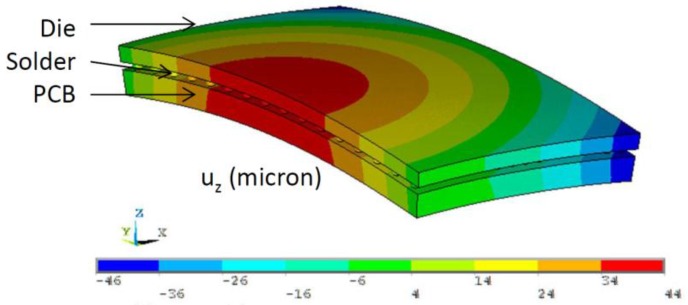
Deformed shape of global model.

**Figure 11:**
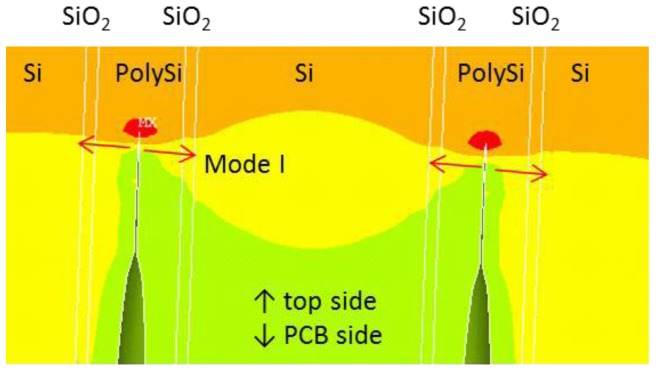
Illustration of the opening mode in poly-Si TSV.

A 3-D board-level FEA model was built for poly-Si TSV. Material properties used for the FEA model are shown in Table 1. We applied fracture mechanics in FEA to study the potential reliability risk for this TSV type. Sub-modeling techniques were used in simulation. The global model of the die-solder-board (or flex) assembly was used to obtain the deformation as well as the stress of the die; for example, Figure 10 shows the deformation with 0.8 mm PCB thickness per 100 °C uniform thermal loading. Stress-free condition was assumed to be at high temperature. A refined local model was made of a region near the die center that contains only one TSV and its surrounding die material (Figure 11). The via, void and crack were constructed in detail in the 3-D local model. Crack was built at the tip of the seam void in the poly-silicon, and the crack surfaces were assumed to have the identical location when there was no external loading applied. The displacement result from the global model was applied to the local model as loading condition, and strain energy release rate (SERR, G) was calculated using virtual crack closure method (VCCM) [36] as output. Then the SERR was converted to stress intensity factor (SIF, K) [37]. The conversion between the G and K is dependent on whether it is plane-stress or plane strain. Though in this case, the stress condition is neither plane-stress nor plane-strain, we selected the one that gave more conservative results for engineering purpose. Because the external loading is mainly normal stress due to the bending of the die, the opening of the crack (mode I) in radial direction was expected to be the major mode (Figure 11); and the effect of sliding mode, shearing mode, and the contribution of hoop stress is much smaller in this case. This was also confirmed by the simulation results.

## 7. Discussion: Reliability Risks of Poly-Si TSV in Sensor Assembled on PCB

### 7.1. Effect of Crack Length and Crack Tip Location

**Figure 12:**
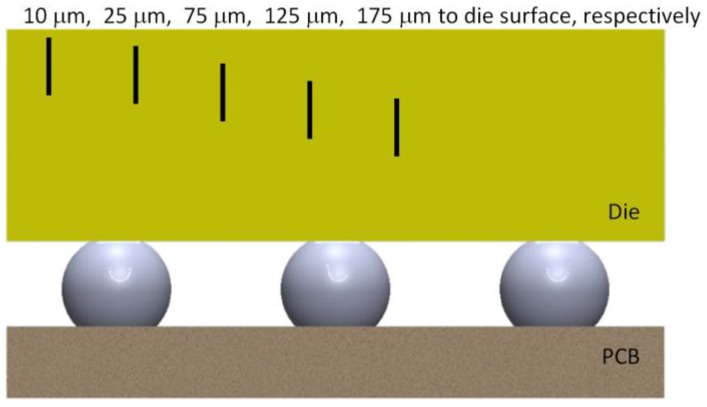
Schematic showing different crack locations.

**Figure 13:**
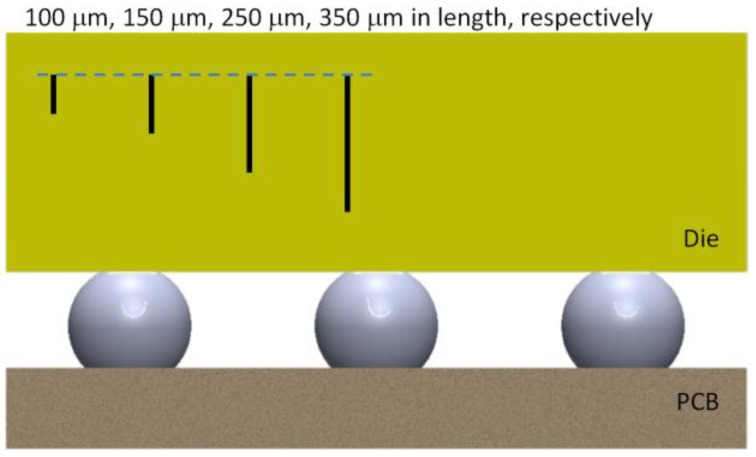
Schematic showing different crack lengths.

**Figure 14:**
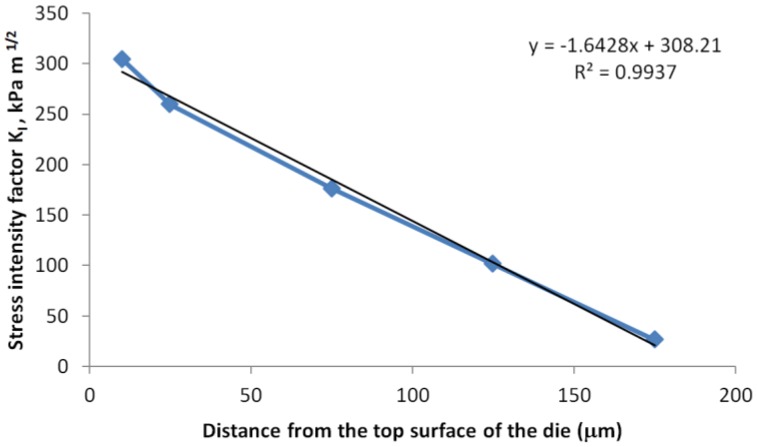
Effect of crack location on stress intensity factor.

The test matrix shown in Table 2 is used to study the effect of crack size and location, and in all the simulation cases in Table 2, the PCB thickness was set as 0.8 mm. Figure 12 and Figure 13 show the crack length and location for each simulation case schematically. To study the effect of crack location, the length of the crack (including the seam void and the tips) was fixed as 150 μm, and the results per every 100 °C thermal loading were compared in Figure 14. There is a linear correlation between the stress intensity factor and the crack location. Because the assembly is always done at high temperature, we assume stress-free condition at high temperature; therefore, the top side of the die experienced tensile stress, and the stress state is linearly related with the coordinate in thickness direction, which explains the linear relation between the SIF and the location of the “top” crack tip that is close to the optical surface.

**Figure 15:**
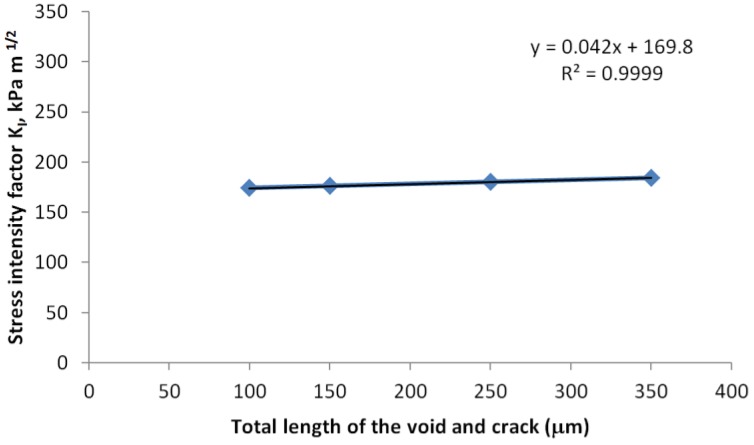
Effect of crack lengths on intensity factor.

However, the crack length has little effect on the SIF if the distance between the crack tip and optical surface of the die remains the same (Figure 15). This also confirms that the reliability risk is directly determined by normal stress due to die bending; therefore, controlling the location of the crack tip is more important than controlling crack length.

### 7.2. Effect of Die and Board Thickness

The thickness of the die and PCB directly determines the warpage and stress of the assembly. Because the linear relation between die stress and SIF at the crack tip has been found in this study (Figure 14), we only need to study the die stress using the global model (without any crack), and the test matrix for this study was shown in Table 3. To study the effect of die thickness, the PCB thickness was set as 0.8 mm, and to study the effect of PCB thickness, the original die thickness was kept unchanged.

**Figure 16:**
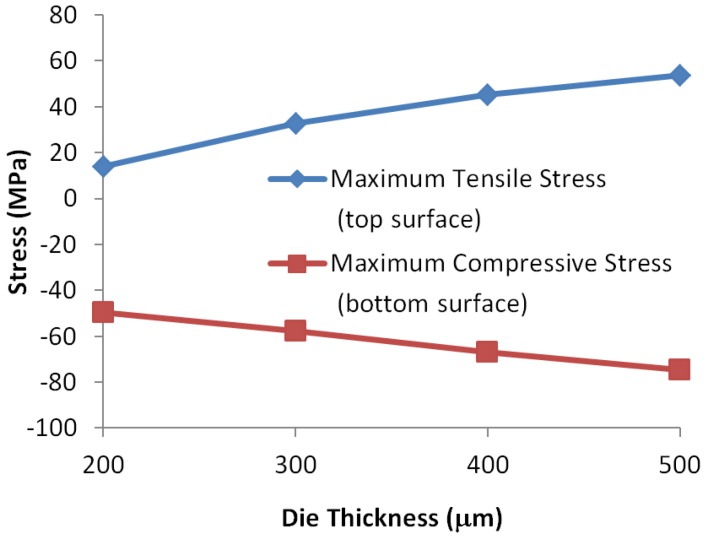
Effect of different die thickness on the maximum stress at die surfaces.

**Figure 17:**
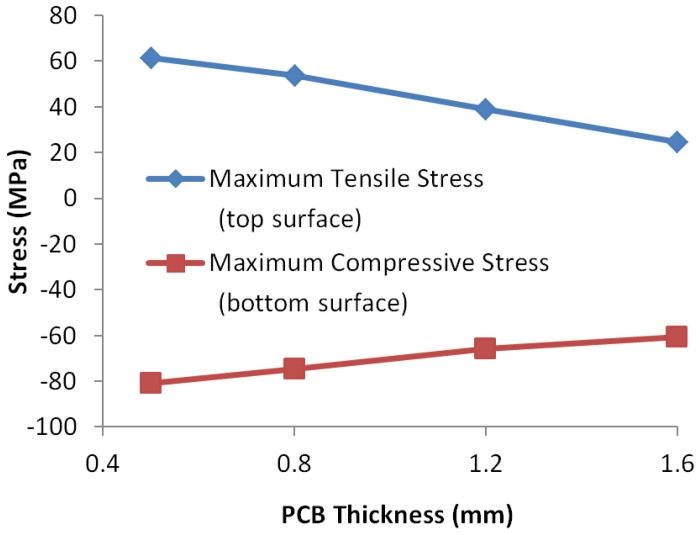
Effect of different PCB thickness on the maximum stress at die surfaces.

For the effect of die thickness, we only considered thickness reduction in this study because 500 μm die is very thick for TSV fabrication. The results for die thickness and PCB thickness are shown in Figure 16 and Figure 17, respectively. Because the bending stress changes linearly in the thickness direction, we plotted the stress at two die surfaces so that the in-plane stress at any point can be calculated using linear interpolation.

Reduction of die thickness significantly reduces the stress on the die, especially the tensile stress on the top surface. In addition, considering that the length of the crack/void will reduce simultaneously with the die thickness and assuming that the distance from the crack tip to die surface can be kept the same, a thinner die would be more preferable because the stress at the same height as the crack tip would be much smaller. If the die thickness does not change, assembling to thicker PCB is preferable.

### 7.3. Possibility of Failure Due to Die Bending

The fracture toughness of poly Si is about 0.84 to 1.25 MPa m^1/2^ [38]. In our simulation cases above, the linear elastic material properties were used and 100 °C thermal loading was applied. Therefore, for other loading cases, we may simply multiply a factor to calculate the SIF. The SnAgCu (SAC) solder materials are most commonly used in the industry. The melting point for SAC305 is about 217 °C. Because of the viscoplastic behavior of the solder alloy, the “stress-free” temperature for the warpage of the die is even lower. Assuming the lowest working temperature for the sensor is 40 °C, the maximum temperature loading could not possibly exceed 260 °C. By rescaling the SIF for this loading level, the SIF can marginally reach the critical condition only at the worst combination, 500 μm die, 0.8 mm PCB, and most importantly, a crack tip that is very close to the die surface. However, this can be prevented by controlling the filling process.

## 8. Discussion: Reliability Risks of Poly-Si TSV in Sensor Assembled on Flexible Substrate

Compared with PCB, the flexible circuit has advantages such as flexibility, light weight, space-saving, etc. It also has been widely used in consumer electronics such as laptop computers and digital cameras. In this study, we also considered assembling the optical sensor to a flexible substrate that contains a polyimide (PI) layer of 25 μm in the middle and two layers of Cu on the top and bottom, each having a thickness of 12 μm. Small gold bumps were used to connect the chip to the flexible substrate. Compared with using a 0.8 mm PCB, the SIF for chip-on-flex assembly is only 23% of the PCB assembly for the same level of thermal load. It is worth noting that the peak temperature of assembling gold bumps using thermo-compression bonding is about 400 °C, which is higher than using reflow soldering for PCB assembly. However, the SIF at the crack tip for chip-on-flex assembly is still smaller even if the largest possible thermal loading is applied.

### Parametric Study on Die Thickness and Substrate Thickness

Two different scenarios were considered for parametric study. The first one reduces the thickness of the die to 300 μm, and the second one doubles the thickness of the PI layer while keeping other parameters unchanged. The first design leads to higher bending stress for the die, which is different from PCB assembly (Table 4 and Table 5). The second design has tensile bending stress on the die as 15.4 MPa and stress intensity factor KI as 75.6 kPam^1/2^, which are higher than those of the original design as well. This difference indicates that the stress is dependent on both the CTE and relative comparison of the modulus of the materials. Theoretical work on this phenomenon can be traced back to Timoshenko’s analysis on bi-material thermostats [39], and later Suhir [40,41], Mishkevich [42], Tsai [43], etc. They studied stress and warpage on die-substrate assemblies in electronic packaging applications. The values for the calculated SIF are still in the safe range for both scenarios.

## 9. Summary

The reliability risks of two different TSVs in different applications were studied. For Cu TSVs used in MEMS packages, we quantitatively compared the effect of different material properties of the filling polymer on the thermo-mechanical stress at critical locations. Results showed that the upper corner of the TSV, which was the joint location of the side wall and the trace that connects to the solder bump, was usually more sensitive to the properties of the filling materials. Generally, smaller CTE and Young’s modulus reduce the stress at the inner corners. However, in reality, many low CTE materials may come with higher modulus and vice versa. In addition, the stress at different locations may have different trends as the geometry or material changes, especially for TSV with a complex structure, so the most critical location might be changeable. Therefore, it is quite necessary to evaluate each structural design and candidate material case by case. For the TSV filled by poly-Si for optical sensor, we used the fracture mechanics approach to assess the reliability risk due to the voids inside the vias. The tip of the seam void was found to be the major concern for board-level reliability. Two kinds of materials for the board were considered: PCB and flexible substrate (polyimide film). Various crack lengths and different crack locations were modeled with the package mounted on PCB. It was found that SIF value increases as the crack tip is closer to the die surface. Changing crack length only did not contribute to a different SIF value. Assuming the failure criteria was the fracture toughness of poly-Si, VCCM indicated that the maximum temperature loading (temperature change) was not allowed to exceed 260 °C. Parametric study on the effects of die thickness and board thickness on board-level reliability was also investigated, for PCB and flexible substrate respectively. For packages on PCBs, a larger die thickness or a smaller PCB thickness caused a larger die stress. When using flexible substrates (with much smaller modules and larger CTE) instead of PCBs, die stress and SIF value were reduced. Furthermore, a smaller die thickness resulted in a larger die stress and SIF value, and a larger flexible substrate thickness led to a larger die stress. Both of these results showed a different trend of die stress from packages on PCBs. However, the calculated SIF values were still in the safe range for both scenarios.

## Figures and Tables

**Table 1:** Material Properties for FEA models.

Material	Young’s Modulus (GPa)	Poisson’s Ratio	CTE (ppm/°C)
Silicon	$E_{x}$ = $E_{y}$ = 169; $E_{z}$ = 130 $G_{\mathrm{xy}}$ = 50.8; $G_{\mathrm{yz}}$ = $G_{\mathrm{xz}}$ = 79.5	$\nu_{\mathrm{xy}}$ = 0.062 $\nu_{\mathrm{yz}}$ = $\nu_{\mathrm{xz}}$ = 0.36	2.6
SiO_2_	75	0.17	0.5
Al	69	0.35	22.2
Copper	110	0.35	16.4
Insulation polymer (Type A)	2.3 to 9.2	0.26	25 to 100
FR4 (Type B)	22	0.28	18
Au (Type B)	2.5	0.42	14.4
Polyimide film (Type B)	2.5	0.34	20 (20 °C); 30 (100 °C); 40 (150 °C)

**Table 2:** Test matrix for studying the effects of crack location and crack length.

Test Matrix		Total Length of Voids and Cracks (μm)			
		100	150	250	350
Crack tip location (distance to die surface, μm)	10		√		
	25		√		
	75	√	√	√	√
	125		√		
	175		√		

**Table 3:** Test matrix for studying the effect of die and PCB thickness.

Test Matrix		Die Thickness (μm)			
		200	300	400	500
PCB thickness (mm)	0.5				√
	0.8	√	√	√	√
	1.2				√
	1.6				√

**Table 4:** Comparison between PCB and flexible on board-level thermo-mechanical reliability (die thickness = 0.5 mm, ΔT = 100 °C).

Parameter	PCB (0.8 mm)	Flexible Substrate (49 μm)
Tensile bending stress on the die (MPa)	53.7	14.2
Stress-intensity factor KI (kPam^1/2^)	304.4	69.3

**Table 5:** Influence of die thickness on thermo-mechanical reliability for packages with flexible substrate (ΔT = 100 °C).

Parameter	Die Thickness (μm)		
	500	400	300
Tensile bending stress on the die (MPa)	14.2	18.2	24.9
Stress-intensity factor KI (kPam^1/2^)	69.3	89.2	120.2
